# Estrogens, Estrogen Receptors Effects on Cardiac and Skeletal Muscle Mitochondria

**DOI:** 10.3389/fendo.2019.00557

**Published:** 2019-08-14

**Authors:** Renée Ventura-Clapier, Jérôme Piquereau, Vladimir Veksler, Anne Garnier

**Affiliations:** Signaling and Cardiovascular Pathophysiology Inserm UMR-S 1180, Université Paris-Sud, Châtenay-Malabry, France

**Keywords:** estrogens, estrogen receptors, mitochondria, heart, skeletal muscle, sexual dimorphism

## Abstract

Mitochondria are unique organelles present in almost all cell types. They are involved not only in the supply of energy to the host cell, but also in multiple biochemical and biological processes like calcium homeostasis, production, and regulation of reactive oxygen species (ROS), pH control, or cell death. The importance of mitochondria in cell biology and pathology is increasingly recognized. Being maternally inherited, mitochondria exhibit a tissue-specificity, because most of the mitochondrial proteins are encoded by the nuclear genome. This renders them exquisitely well-adapted to the physiology of the host cell. It is thus not surprising that mitochondria show a sexual dimorphism and that they are also prone to the influence of sex chromosomes and sex hormones. Estrogens affect mitochondria through multiple processes involving membrane and nuclear estrogen receptors (ERs) as well as more direct effects. Moreover, estrogen receptors have been identified within mitochondria. The effects of estrogens on mitochondria comprise protein content and specific activity of mitochondrial proteins, phospholipid content of membranes, oxidant and anti-oxidant capacities, oxidative phosphorylation, and calcium retention capacities. Herein we will briefly review the life cycle and functions of mitochondria, the importance of estrogen receptors and the effects of estrogens on heart and skeletal muscle mitochondria.

## Cardiac and Skeletal Muscle Mitochondria

### Mitochondria

#### Origin and Life Cycle

Mitochondria are small intracellular organelles of approximately one micron in size that are present in granular or filamentary form and in variable number from one cell type to another. Mitochondria derive from aerobic α-proteobacteria, which were engulfed by anaerobic archeobacteria giving rise to primitive eukaryotic cells a few billion years ago. The host cell provides the necessary substrates for the mitochondria that metabolize these substrates and provide ATP to the host cell through oxidative phosphorylations; in turn, mitochondria attenuate intracellular concentration of oxygen, which is a potentially toxic substance for cellular constituents.

Due to their ancient bacterial origin, mitochondria are surrounded by two membranes, an outer membrane, and an inner membrane exhibiting a particular lipid composition (rich in cardiolipin) and numerous invaginations or ridges, delimiting two spaces: the intermembrane space and the matrix space. The matrix of each mitochondrion contains several copies of a circular DNA (mtDNA) of 16 kb in mammals encoding some proteins as well as the RNAs required for the synthesis of these proteins. During evolution, mitochondrial DNA retained only 13 genes coding for subunits of the respiratory chain, two ribosomal RNA, and 22 transfer RNAs. Most of the mitochondrial genes have been transferred to the nucleus of the host cell. In mammals, the mitochondrial proteome is composed of more than a thousand of proteins (1). After fertilization, the mitochondria are of maternal inheritance. However, the fact that the vast majority of mitochondrial proteins are of nuclear origin underlies the tissue- and sex-specificity of mitochondria.

**Figure 1:**
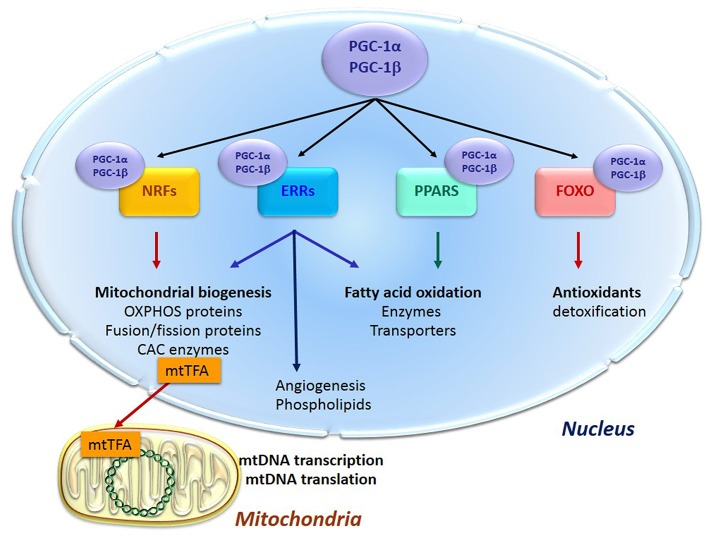
Transcription cascade of mitochondrial biogenesis. Increased expression or activity of the master regulators of mitochondrial biogenesis “peroxisome proliferator activator receptor (PPAR) γ co-activators” (PGC-1s) α and β activate the expression of the nuclear respiratory factors 1 and 2 (NRFs) which induce the expression of the mitochondrial transcription factor A (mtTFA). The latter translocates to mitochondria, binds to mtDNA and activates its transcription and replication. PGC-1s also activate other transcription factors such as PPARα, mainly regulating lipid metabolism, estrogen-related receptors (ERRs) involved in substrate metabolism, energy transfer, angiogenesis and detoxification of reactive oxygen species, and the forkhead family of transcription factors (FOXOs) that activate antioxidant and detoxifying proteins.

Energy supply and cell survival depend on the mitochondrial life cycle, which includes biogenesis, dynamics, and elimination of defective organelles by mitophagy. Mitochondrial proliferation consists of growing of preexisting organelles, through a process called mitochondrial biogenesis followed by mitochondrial division (fission). This biogenesis requires the coordination of both the nuclear and the mitochondrial genomes; it is mostly under the control of the nucleus through transcriptional cascades involving coactivators and specific transcription factors (Figure 1). Among the most important are (1) the family of the transcriptional coactivators “peroxisome proliferator activator receptor (PPAR) γ co-activators” (PGC-1s) α and β, the master regulators of mitochondrial biogenesis, (2) nuclear respiratory factors NRF1 and two involved in the nuclear control of mitochondrial biogenesis (2), (3) transcription factors such as PPARα, mainly regulating lipid metabolism, (4) estrogen-related receptors (ERRs) involved in substrate metabolism, energy transfer, angiogenesis, and detoxification of reactive oxygen species and (5) the mitochondrial transcription factor A (mtTFA) which once synthesized, migrates to the mitochondrial matrix where it activates the transcription and replication of mtDNA.

An important step in the mitochondrial life cycle is the fission of mother mitochondria into two daughter mitochondria, which is coordinated by specialized GTPases. In most cells, mitochondria are organized into a network that undergoes frequent fusion and fission events. This process is called mitochondrial dynamics. Fission is dependent on the DRP1 protein and its docking proteins, fission protein 1 (Fis1) and mitochondrial fission factor (MFF). Fusion is controlled by mitofusins (MFN1 and 2) for the outer membrane and optic atrophy 1 (OPA1) for the inner membrane. These fusion/fission processes are very slow in the adult cardiomyocyte under normal conditions (3).

Finally, dysfunctional mitochondria are eliminated through a specific mechanism of autophagy called mitophagy. Autophagy is the process by which cellular components such as macromolecules and organelles are recycled, allowing for the elimination of cellular waste and the reconstitution of nutrient stores. Mitophagy is the specific process for elimination of mitochondria and it is involved in quality control and mitochondrial turnover rate regulation. This process involves many proteins, among which the E3-ubiquitin ligase Parkinson juvenile disease protein 2 (PARKIN) and the PTEN-induced putative kinase 1 (PINK1) can be mentioned, allowing the identification of damaged mitochondria and their addressing to the autophagosome, the latter subsequently fusing with the lysosome to form autophagolysosomes.

#### Mitochondrial Functions and Regulation

Mitochondria are the main site of transformation of the energy of different substrates into chemical energy in the form of ATP. ATP consumption is extremely high in the heart where mitochondria provide more than 90% of the energy needed for contraction and cell pumps. The linear relationship between oxygen consumption and cardiac work shows that the mitochondria work in a “*pay as you go*” manner in this tissue. The heart can use a variety of substrates to regenerate ATP according to their availability, mainly fatty acids. While slow oxidative muscles also largely depend on oxidative metabolism for ATP production and uses fatty acids and glucose, fast skeletal muscles function in a “*work first pay later*” manner and preferably use glucose, glycogen and glycerol-3P. Lipid oxidation is responsible for 60 and 80% of totally consumed oxygen by soleus and cardiac muscle mitochondria, respectively, but for only a few percent by glycolytic gastrocnemius muscle mitochondria. On the opposite, glycerol-3-P utilization represents more than 80% of substrate used by gastrocnemius muscle mitochondria, while <10% of substrates used by cardiac mitochondria (4).

The common degradation product of lipids, amino acids and carbohydrates in mitochondria is acetyl-CoA, which enters the Krebs cycle in the mitochondrial matrix resulting in the production of reducing equivalents (NADH and FADH_2_). These reducing equivalents are re-oxidized by the electron transport chain (ETC), also called respiratory chain, through the process of oxidative phosphorylations (OXPHOS). The respiratory chain is composed of four complexes forming a redox chain which results in the reduction of molecular oxygen to water and the creation of a proton gradient across the mitochondrial inner membrane. This electrochemical gradient of protons serves as a proton-motive force to rephosphorylate ATP from ADP by ATP synthase considered as complex V of ETC. Mitochondrial respiration is controlled by ADP according to a Michaelis/Menten relationship (5) and is modulated by calcium through the regulation of the activity of certain enzymes in the Krebs cycle and the respiratory chain (6). ATP is exchanged for ADP by the adenine nucleotide translocator anchored in the inner membrane. In muscle and cardiac cells, most of the ATP produced is immediately transformed in the inter-membrane space into phosphocreatine (PCr) by mitochondrial creatine kinase (CK) located on the outer surface of the inner membrane in the reaction:

$$\begin{array}{l} \text{ATP}+\text{creatine}\Leftrightarrow \text{ADP}+\text{PCr}+\;{\text{H}}^{+}. \end{array}$$

The newly formed PCr is channeled to the sites of energy utilization by the cytosolic CKs and allows the rephosphorylation of ADP locally produced by the ATPases (5, 7).

In addition to providing the greatest amount of energy to the host cell, mitochondria participate in other cellular functions such as ionic homeostasis among which calcium plays a pivotal role, production and regulation of reactive oxygen species (ROS), pH control and cell death. Besides, mitochondria are crucial for steroid hormone synthesis, thermogenesis, and lipid and carbohydrate utilization in a tissue-specific manner.

Mitochondria are the main site of ROS production in the cells and also the primary target of oxidative damages. ROS are mainly produced at the level of complex I and complex III of the respiratory chain. In physiological conditions, mitochondrial and cytosolic anti-oxidant defenses allow to control any excess of ROS production. Dysregulation of ROS production is involved in many pathological situations and in aging.

Another critical factor in mitochondrial function is the regulation of intracellular and intramitochondrial calcium. Although calcium is known to activate mitochondrial dehydrogenases, when present in excess, it uncouples mitochondria and decreases ATP production. High calcium collapses the mitochondrial membrane potential and induces the opening of the permeability transition pore (PTP), triggering the liberation of proapoptotic factors and leading to cell death (8).

#### Tissue Specificity of Mitochondria

Quantity, composition and function of mitochondria are adapted to the tissue in which they are embedded (9). It is generally believed that tissues with high and sustained ATP demand like striated muscles depend on oxidative phosphorylations ensured by the mitochondria. However, striking differences exist between oxidative cardiac and skeletal muscles on one hand and glycolytic skeletal muscles on the other hand. Cardiac muscle and postural skeletal muscles are strictly dependent on oxidative ATP production as they have to sustain long-term contractile activity. Oxidative muscles should have accurate adjustment of energy production to energy consumption. This is different from fast skeletal muscles whose contractile activity is rapid and short in duration and is mainly supported by ATP and PCr stores and glucose- or glycogen-driven ATP production.

In the myocardium, one of the most oxidative tissues of the body, mitochondria occupy 30–40% of the cell volume against 6–8% in the slow and 2–3% in the fast muscle fibers. The mitochondria in oxidative tissues like the heart and slow oxidative postural skeletal muscles are more numerous, located largely near the myofilaments, and able to use essentially fatty acids. They have a relatively low sensitivity to ADP and a significant amount of mitochondrial CK allowing them to participate in intracellular energy transfer shuttles. They are adapted to the continuous supply of energy, necessary for muscular endurance work and cardiac function (10).

In fast skeletal muscles, mitochondria are less numerous, with a high sensitivity to cytosolic ADP (11) and are more dependent on glycolysis-derived substrates such as pyruvate and glycerol-3P (4). The energy required for the phasic activity of fast muscles is in fact essentially dependent on anaerobic glycolysis; energy metabolism reflects rapid and short-term use of large amounts of cytosolic ATP and PCr. Importantly, the CK system also exhibits important tissue specificity among striated muscles (9).

### Sexual Dimorphism of Mitochondria

Although mitochondria are of maternal inheritance in both males and females, it should be kept in mind that, as discussed above, almost all mitochondrial proteins are encoded by the nucleus and thus under the influence of sex chromosomes, epigenetic marks and importantly, circulating sexual hormones. It is thus not surprising that in addition to a clear tissue specificity of mitochondria, a sexual dimorphism of mitochondria has been observed and starts to be studied in further details. Evidence is accumulating that this dimorphism takes part in the sex-specificity of important pathologies (12–14). Mechanistic pathways involved in sex differences in cardiovascular diseases has been recently reviewed in depth (15).

#### Cardiac Muscle

Gene expression profiling of mouse and human cardiac samples revealed sex and aged dependent expression patterns (16, 17). Among the differences, energy metabolism pathways are well-represented. However, it is generally accepted that cardiac oxidative capacity does not essentially differ between males and females (18–20) except for fatty acid utilization capacity that seems to be higher in young females, and for oxidative capacity that declines slower with age in older females (21, 22).

The most striking difference between male and female mitochondria lies in antioxidant defenses (12, 23). Female cardiac mitochondria seem to produce less ROS (18, 20) although this may be species or strain-dependent (19). Decreased ROS generation in female rat cardiac mitochondria has been attributed to posttranslational modifications of mitochondrial proteins involved in ROS handling (24).

Cardiac mitochondria of female rats have a greater calcium retention capacity than the male ones (25). Calcium kinetics also differ between male and female rat cardiac mitochondria (26). Mitochondria from females are more resistant to mitochondrial swelling at high calcium concentration (27). The lower Ca^2+^ uptake rates and the maintenance of mitochondrial membrane potential under conditions of high Ca^2+^ in female rat cardiac mitochondria have been attributed to modulation of the calcium uniporter (28).

Thus, sexual dimorphism of cardiac mitochondria involves a lower production of ROS, higher antioxidant defenses, and a better regulation of intra-mitochondrial calcium; all of which participate in the better resistance of female cardiomyocytes to ischemia/reperfusion injury, heart failure or cardiotoxicity.

#### Skeletal Muscle

It is well-established that female rodents have much higher endurance capacities than their male counterparts (29, 30). Interestingly woman skeletal muscle demonstrates an increased fatigue resistance compared to man muscle (31). Endurance capacity is highly dependent on skeletal muscle mitochondrial content and quality, as well as capacity to oxidize lipid substrates. Compared to males, females rely to a greater extent on fat oxidation during exercise (32) and skeletal muscles of women have higher intracellular lipid content than male muscles (33). Moreover, gastrocnemius muscle of female rats exhibits higher mitochondrial mass, oxidative-phosphorylative capacities, and mtDNA/nDNA compared to males (34, 35).

Different lean mass/body mass ratio in male and females, and the plasticity of skeletal muscle mitochondria in response to exercise training and exercise capacity (36), make comparison of skeletal muscle from men and women more difficult to interpret than for laboratory animals which are usually kept in standard low activity conditions. Recent studies have brought new knowledge on sex differences in human skeletal muscle mitochondria (37, 38). Whether these differences are due to differences in skeletal muscle composition in terms of oxidative vs. glycolytic fibers, remain to be determined. In young men and women of similar aerobic exercise capacity, a higher mitochondrial volume density and increased capacity for fatty acid and lactate oxidation were found in women skeletal muscle (39) thereby suggesting real differences between men and women mitochondrial properties.

Other aspects of sex differences in mitochondria include resistance to calcium overload, generation of radical oxygen species, and susceptibility to PTP opening and apoptosis. Only few data are available for skeletal muscle.

Thus, mitochondria of skeletal muscle also seem to exhibit a sexual dimorphism which could explain the higher facility of females to adapt to altered metabolic energy situations. However, data on skeletal muscle are so far sparse and undoubtedly, sexual dimorphism should be investigated more systematically in order to know whether skeletal muscle mitochondria contribute to different men and women muscle function and susceptibility to pathologies (13).

How much of these effects could be attributed to sex hormones and more specifically to estrogens and estrogen receptors will be discussed below.

## Estrogen Receptors and Mitochondria

**Figure 2:**
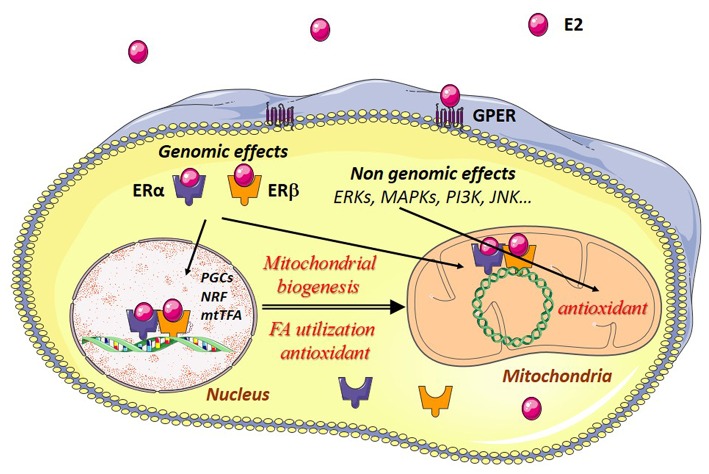
Estrogen effects in muscle cells. Estradiol (E2) binds to its cytosolic receptors, estrogen-receptor α (ERα) and β (ERβ) inducing the translocation of the complex E2/estrogen receptors (ER) to the nucleus. Interaction of this complex with nuclear DNA results in the transcription of *Pgc-1*α*, Nrfs*, and other mitochondrial genes are involved in mitochondrial biogenesis, fatty acid utilization, and antioxidant defenses. Estradiol also interacts with ERs bound to mitochondrial DNA (mtDNA), thereby leading to transcription and replication of the mtDNA. E2 can also bind to G-protein-coupled estrogen receptors (GPER) at the plasma membrane, activating intracellular signaling pathways like extracellular signal-regulated kinase 1 and 2 (ERK1/2), p38 mitogen-activated protein kinases (MAPKs), phosphoinositide 3-kinase (PI3K), c-Jun-NH2-terminal protein kinase (JNK), and others. This results in the transcription of genes encoding antioxidant enzymes, thus reinforcing in particular the antioxidant defenses of the mitochondria.

Estrogen effects are mainly mediated through estrogen receptors (ERs). ERs belong to the family of steroid hormone receptors themselves members of the family of ligand-activated transcription factors. Three estrogen-receptors have been described, estrogen-receptor alpha (ERα), estrogen receptor beta (ERβ), and the G-protein coupled estrogen receptor (GPER) with specific tissue and intracellular locations (Figure 2). These receptors are thought to be present in practically all cell types in varying quantities. Their regulation and their location in striated muscles are still not well-understood.

Estrogens may have genomic and non-genomic effects. ERs may function as nuclear receptors and transcription factors in the nucleus and as signaling molecules in the plasma membrane. Estrogens regulate nuclear gene transcription by binding and activating the classical genomic estrogen receptors α and β (ERα and ERβ) and by activating plasma membrane-associated ERs, and GPER. The major circulating estrogen is 17β-estradiol (E2) which equally binds ERα and ERβ. The genomic effects involve dimerization of two ERs upon binding of E2 which then translocate to the nucleus and bind to estrogen response elements (ERE), thus regulating specific gene expression. Binding of E2/ERα complex to estrogen response elements (EREs) is regulated by phosphorylation independent of ligand binding. For detailed description of the genomic effects of E2/ER see (40).

Non-genomic effects involve rapid, within seconds or minutes, signaling actions through activation of membrane-associated ERs and GPER and subsequent activation of signaling pathways like extracellular signal-regulated kinase 1 and 2 (ERK1/2), increased phosphorylation of c-Jun-NH2-terminal protein kinase (JNK), phosphoinositide 3-kinase (PI3K), protein kinase B (PKB), glycogen synthase kinase3β (GSK3β), β-catenin, calcineurin, mechanistic target of rapamycin (mTOR), p38 mitogen-activated protein kinase (MAPK), and others (15, 41).

Both ERα and ERβ were found in male and female cardiomyocytes (42, 43). However, one study showed transcripts only for ERα in isolated cardiomyocytes from neonatal or adult rats suggesting that ERβ could be present in other cardiac cell types like fibroblasts or vascular cells (44). The presence of both ERs in cardiomyocytes is thus a matter of debate while skeletal muscles express both ERs with certainty (45, 46).

In addition to their presence in nuclei and plasma membrane, both ERα and ERβ have been detected in mitochondria of many cell types and species [for details see recent reviews (40, 47–49)]. However, ERβ seems to be the main estrogen receptor present in mitochondria as demonstrated by immunohistochemistry, immunocytochemistry, and immunoblots using a large panel of antibodies and mechanisms of import have been studied (see (50) for review).

Mitochondrial location of E2 receptors seems to be anyway tissue-specific. The presence of ERβ was detected in human heart mitochondrial proteins by Yang et al in 2004 (51) while not detected in liver cells by the same methods (MALDITOF) (52). ERβ was also identified in the skeletal muscle tissue and the C2C12 skeletal muscle cell line (53, 54). In this cell line, ERβ interacts with the chaperone HSP27 in the mitochondria and stabilizes it (55). However, additional experiments are needed to clearly identify the tissue specific expression and role of the different estrogen receptors in mitochondria.

## Estrogen Effects on Mitochondria

The effects of estrogens on mitochondria are not restricted to their presence and role inside mitochondria. As discussed above, expression of mitochondrial proteins is mainly controlled by nuclear genome so that nucleus controls mitochondrial biogenesis. It is thus not surprising that the major effects of estrogen on mitochondria arise from their nuclear effects.

Estrogens exert a protective role on mitochondria by direct and indirect effects in a number of tissues [reviewed in (40, 48)]. Estrogen regulation of mitochondrial mass and function has been shown to participate in vascular, cardiac and neuronal protection (56–59). Estrogens appear to modulate various aspects of mitochondrial function including ATP production, ROS generation, antioxidant defenses, mitochondrial membrane potential, and calcium handling (50). Moreover, estrogens via ERs are involved in life cycle of mitochondria controlling mitochondrial biogenesis, mitochondrial quality control, and mitophagy. These effects may be mediated by both genomic and non-genomic effects.

Estrogens favor mitochondrial biogenesis in a tissue-specific manner by stimulating or inhibiting the expression of mitochondrial proteins from both nuclear and mitochondrial genomes (48, 60). Estrogens directly modulate the expression of mitochondrial protein genes by binding of ER/E2 to the ERE of metabolic genes in the nucleus. They increase the expression of the master regulator of energy metabolism and mitochondrial biogenesis PGC-1α and its downstream targets (61, 62). E2 also stimulates mitochondrial biogenesis by ER/E2 mediated activation of the transcription factors NRFs (56, 62, 63). NRF1 increases transcription of mitochondrial nuclear-encoded genes and of mitochondria-encoded genes through increased production of the transcription factor mtTFA (64). This is mediated by ERα and by the presence of an ERE in the promoter of the NRFs which can bind both ERα and ERβ in an estrogen-dependent manner (63).

ERs may also bind to mitochondrial DNA and are involved in the E2-induced expression of mtDNA and respiratory chain proteins, suggesting a role for these receptors in the regulation of mtDNA transcription and replication. The presence of ERs and their effects on both the nuclear and mitochondrial genomes would ensure their coordination in response to estrogens.

Whether ERα and ERβ exert similar or dissimilar roles in a tissue-specific manner is an additional level of complexity. Indeed, in addition to their subcellular localization, estrogen action depends on the expression and content of selective estrogen receptor modulators (SERMs). These SERMs induce various conformational changes in the ERs and differential recruitments of corepressors, coactivators, and transcription factors so that they can be either full or partial agonist or antagonists (47).

Thus, E2 is able to directly or indirectly modulate mitochondrial biogenesis and mitochondrial properties. The implication and role of ERα and/or ERβ may be tissue specific.

### Estrogen Effects on Cardiac Mitochondria

Several studies have described the effects of ovariectomy on cardiac mitochondria. Quantitative proteomic analysis of cardiac mitochondria from ovariectomized (OVX) aged rats revealed a remarkable reduction in mitochondrial proteins involved in ETC, oxidative phosphorylation, lipid, and carbohydrate metabolism as well as oxidative stress and apoptosis (65). Ovariectomy decreases the activity of ETC complexes, increases mitochondrial membrane lipid peroxidation, and decreases calcium handling capacity (66). It decreases complex I-driven ATP synthesis and increases stress-induced ROS production of isolated cardiac mitochondria (58). Ovariectomy also downregulates cardiac mitochondrial biogenesis and function markers and increases oxidative stress through a possible GPER-mediated effect (67). These effects could be counteracted by E2 treatment. For example, E2 increases NRF-1, TFAM, PGC-1α, and mitochondrial protein levels in the heart of OVX mice (68). Mitochondrial alterations due to E2 deficiency may also be caused through miR-23a-mediated PGC-1α downregulation, which may subsequently be involved in the menopause-associated concentric cardiac hypertrophy (69).

On the other hand, physiological concentrations of estrogens do not affect mitochondrial respiratory functions in normal conditions but protect heart mitochondria from high calcium-induced release of cytochrome *c* (70). Activation of the membrane receptor GPER provides a positive effect after ischemia-reperfusion injury by inhibiting PTP opening, an effect mediated by the ERK pathway (71). E2 via ERβ mediates cardioprotection following trauma hemorrhage by activating the transcriptional cascade of mitochondrial biogenesis (62) and inhibiting the mitochondria-mediated apoptotic pathway (72). ERβ also appears to mediate the anti-apoptotic effects of estrogen in female adult heart following pressure overload (73). In cardiomyocytes exposed to ischemia/reperfusion, estrogens prevent cardiomyocyte apoptosis by inhibiting mitochondrial ROS formation (74). ERβ also mediates the increased expression of key regulators of cardiac mitochondrial function and respiratory chain proteins occurring in female mice following voluntary running (30). Thus, effects of E2 on mitochondria participate in the regulatory effects of estrogens. Finally, cardiomyocyte-specific deletion of ERα induces alterations in expression of some proteins involved in carbohydrate and lipid metabolism (75).

Whatsoever, estrogen actions in the heart seem to be more restricted to pathological states, during which estrogens mediate activation of mitochondrial biogenesis and cellular protection.

### Estrogen Effects on Skeletal Muscle Mitochondria

Skeletal muscle possesses higher plasticity than cardiac muscle and exhibits considerable metabolic flexibility in response to hormonal stimulation, environmental factors, and exercise training. Skeletal muscles represent around 40% of body mass, and as a result they play a crucial role in insulin-sensitivity and glucose regulation.

Ovariectomy induces profound alteration of muscle biology and function. It decreases fatigue resistance, a factor known to depend on mitochondrial mass (76). Skeletal muscle of OVX mice shows lower use of fatty acid substrates, decreased PGC1α expression, reduced mitochondrial content, and increased compensatory extramitochondrial ATP synthesis during exercise, most of which could be reverted by E2 treatment (77). Loss of E2 induces a rapid decline in skeletal muscle mitochondrial respiratory capacity, independently of mitochondrial content. This is likely mediated by changes in complex I function, accompanied by a ~40% decrease in the reduced to oxidized glutathione ratio, effects that could also be reversed by E2 treatment (78).

Deletion of ERα in skeletal muscle has profound metabolic consequences. Muscle-specific ERα knock-out mice (MERKO) exhibit decreased muscle mass, obesity, and insulin resistance with muscle mitochondrial dysfunction, altered mitochondrial dynamics and diminished mitophagy (79). Knock-down of ERα in C2C12 myotubes reduces oxygen consumption rates and increased production of ROS thus promoting cellular oxidative stress (79). In parallel, the mammalian mitochondrial polymerase *Polg1* is also down-regulated following deletion of ERα, providing an additional mechanism for defective mitochondrial function in ERα deficiency. ERα also seems to affect mitochondrial quality control and dynamics because mitochondria of MERKO mouse muscle are elongated and hyperfused due to shift of the fusion/fission equilibrium toward fusion associated with a deficit in the fission protein DRP1 and increased levels of the fusion proteins OPA1 and MFN2 (79).

Conversely, estrogens are required to maintain muscle function because they have a positive effect on muscle mass, muscle regeneration after injury, and growth of satellite cells in young female mice [reviewed in (80)]. Estrogens regulate levels of enzymes associated with fatty acid metabolism explaining the higher capacity of woman skeletal muscles to utilize fatty acids (81, 82). In muscle cells, E2 plays also a major role in the inhibition of mitochondria-dependent apoptosis (83, 84). Physiological concentrations of E2 abrogate cytochrome *c* release and DNA damage induced by oxidative stress (47).

The beneficial effects of E2 derive from both genomic and non-genomic effects. In C2C12 muscle cells, E2 can interact with ERs located in the cell membrane and mitochondria to promote the activation of ERK, p38 MAPK, and the PI3K/Akt/p-Bad survival cascade thus abrogating the mitochondrial damage induced by hydrogen peroxide (83). E2 stimulates mitochondrial biogenesis in white gastrocnemius from OVX by increasing NRF1 transcript expression (85). Recently another mechanism by which E2 status broadly influences energy homeostasis has been described (78). It is shown that in skeletal muscle, E2 associates to the inner mitochondrial membrane, where it lowers membrane microviscosity, a mechanism that in turn promotes bioenergetic function, cellular redox balance, and insulin sensitivity thus offering a biophysical mechanism by which E2 influences energy homeostasis independently of ERα (78). This newly described mechanism of action of E2 on mitochondria may potentially be operative in other tissues; however, more studies are needed since this effect of E2 does not seem to be present in liver (86).

Importantly, postmenopausal women treated with hormone replacement therapy have substantially reduced relative risk of developing type 2 diabetes (87, 88). E2 exerts antidiabetogenic effects by protecting mitochondrial and cellular redox function in skeletal muscle of OVX mice (78). This insulin-sensitizing effect seems to be mediated by ERα. Estrogens induce insulin-sensitizing effects both by a reduction in the direct effects of ERα on insulin signaling as well as indirect effects of ERα on insulin action mediated by mitochondrial dysfunction (89). Estrogens thus appear to be an important target allowing to correct metabolic dysfunctions by pharmacological means (89, 90).

## Conclusions and Perspectives

Estrogens are important participants in metabolic regulation and mitochondrial function. Mitochondria are involved in a vast range of cellular processes, and their dysfunction is intrinsically associated with chronic diseases. Accumulating evidence suggests that many chronic diseases display gender specificity with females generally exhibiting beneficial metabolic profile. Among possible factors, sex-specificity of mitochondria plays a major role in the sexual dimorphism of chronic pathologies (12). Some of these effects could be mediated by estrogens via estrogen receptors. Whether present in nucleus, in mitochondria or bound to cell membrane these latters exert profound genomic and non-genomic effects on mitochondria. They affect among other things mitochondrial biogenesis, ROS production, calcium homeostasis, and mitochondrial pathway of apoptosis in a tissue-specific manner.

Female sex is known to be associated with a reduced risk of developing cardiovascular diseases when compared to males (15, 91), an effect mediated in large part by estrogen and estrogen receptors. It appears that although sex differences in cardiovascular pathologies and metabolic diseases are being recognized, considerations of this issue in myopathologies are yet underestimated (13). Certainly, knowing the importance of mitochondria in the genesis of myopathologies, the sexual dimorphism of skeletal muscle mitochondria could be a contributing factor (13). However, our understanding of sex differences is still incomplete and highlights the importance of developing mechanistic studies to delineate more precisely estrogen receptor expression, localization, and function in males and female heart and skeletal muscles. It is still distressing that, while we are entering the era of personalized medicine and personalized therapeutic strategies, gender differences are still poorly taken into account in the majority of the experimental and clinical researches.

## Author Contributions

RV-C wrote the first version of the review. VV, AG, and JP participated actively in the writing of the final version.

### Conflict of Interest Statement

The authors declare that the research was conducted in the absence of any commercial or financial relationships that could be construed as a potential conflict of interest. The handling Editor declared a past co-authorship with one of the authors RV-C.

## Abbreviations

CK: creatine kinase
E2: 17β-estradiol
ER: estrogen receptor
ERE: estrogen response element
ERK1/2: extracellular signal-regulated kinase 1 and 2
ERR: estrogen-related receptors (ERRs)
ETC: electron transport chain
Fis1: fission protein 1 (Fis1)
GPER: G-protein coupled estrogen receptor
GSK3β: glycogen synthase kinase3β
JNK: c-Jun-NH2-terminal protein kinase
MERKO: muscle-specific ERα knock-out mice
Mff: mitochondrial fission factor
MFN: mitofusins
mtDNA: mitochondrial DNA
mTOR: mechanistic target of rapamycin
mtTFA: mitochondrial transcription factor A
NRF: nuclear respiratory factors
OPA1: optic atrophy 1
OVX: ovariectomy
OXPHOS: oxidative phosphorylations
p38 MAPK: mitogen-activated protein kinase
PARKIN: Parkinson juvenile disease protein 2
PCr: phosphocreatine
PGC-1: PPAR γ co-activator
PI3K: phosphoinositide 3-kinase
PINK1: PTEN-induced putative kinase 1
PKB: protein kinase B
PPAR: peroxisome proliferator activator receptor (PPAR) γ co-activators
PTP: permeability transition pore
ROS: reactive oxygen species
SERM: selective estrogen receptor modulator.
